# Voxelwise distribution of acute ischemic stroke lesions in patients with newly diagnosed atrial fibrillation: Trigger of arrhythmia or only target of embolism?

**DOI:** 10.1371/journal.pone.0177474

**Published:** 2017-05-24

**Authors:** Timolaos Rizos, Andreas J. Bartsch, Timothy D. Johnson, Felix Dittgen, Thomas E. Nichols, Uwe Malzahn, Roland Veltkamp

**Affiliations:** 1 Department of Neurology, University of Heidelberg, Heidelberg, Germany; 2 Department of Neuroradiology, University of Heidelberg, Heidelberg, Germany; 3 Oxford Centre for Functional MRI of the Brain, University of Oxford, John Radcliffe Hospital, Oxford, United Kingdom; 4 Department of Neuroradiology, University of Würzburg, Würzburg, Germany; 5 Department of Biostatistics, University of Michigan, Ann Arbor, United States of America; 6 Department of Statistics & Warwick Manufacturing Group, University of Warwick, Coventry, United Kingdom; 7 Clinical Trial Center Würzburg, University Hospital Würzburg, Würzburg, Germany; 8 Department of Stroke Medicine, Imperial College London, London, United Kingdom; Fraunhofer Research Institution of Marine Biotechnology, GERMANY

## Abstract

**Objective:**

Atrial fibrillation (AF) is frequently detected after ischemic stroke for the first time, and brain regions involved in autonomic control have been suspected to trigger AF. We examined whether specific brain regions are associated with newly detected AF after ischemic stroke.

**Methods:**

Patients with acute cerebral infarctions on diffusion-weighted magnetic resonance imaging were included in this lesion mapping study. Lesions were mapped and modeled voxelwise using Bayesian Spatial Generalised Linear Mixed Modeling to determine differences in infarct locations between stroke patients with new AF, without AF and with AF already known before the stroke.

**Results:**

582 patients were included (median age 68 years; 63.2% male). AF was present in 109/582 patients [(18.7%); new AF: 39/109 (35.8%), known AF: 70/109 (64.2%)]. AF patients had larger infarct volumes than patients without AF (mean: 29.7 ± 45.8 ml vs. 15.2 ± 35.1 ml; p<0.001). Lesions in AF patients accumulated in the right central middle cerebral artery territory. Increasing stroke size predicted progressive cortical but not pontine and thalamic involvement. Patients with new AF had more frequently lesions in the right insula compared to patients without AF when stroke size was not accounted for, but no specific brain region was more frequently involved after adjustment for infarct volume. Controlled for stroke size, left parietal involvement was less likely for patients with new AF than for those without AF or with known AF.

**Conclusions:**

In the search for brain areas potentially triggering cardiac arrhythmias infarct size should be accounted for. After controlling for infarct size, there is currently no evidence that ischemic stroke lesions of specific brain areas are associated with new AF compared to patients without AF. This challenges the neurogenic hypothesis of AF according to which a relevant proportion of new AF is triggered by ischemic brain lesions of particular locations.

## Introduction

In up to 25% of patients with ischemic stroke or transient ischemic attacks (TIA) atrial fibrillation (AF) is newly detected after the event [1], and it has been suggested that the detection of AF immediately after a stroke may represent the cardiac consequence rather than the cause of cerebral ischemia (i.e., “neurogenic AF” hypothesis) [2].

Lesions in specific areas of the brain–especially those involved in autonomic control–may incite cardiac dysregulations [2–9]. In particular, the insula is believed to play a crucial role in such post-stroke autonomic dysregulation. Insular infarcts have been associated with decreased heart rate variability, increased incidence of sudden death [10,11], increased Troponin levels [4], and various electrocardiographic abnormalities [3,7–9]. However, the insula also represents a frequent target of cardiogenic embolism in the middle cerebral artery (MCA) territory [5,11], and MCA infarctions including the insula are often larger than strokes in other vascular territories [12]. Consequently, it remains unclear whether a poststroke arrhythmia that is newly detected after the ischemic event is of specific neurogenic origin or has already been present before and has actually caused the stroke. This is particularly relevant in the case of AF because it is unknown whether AF that is newly diagnosed after stroke carries a similar risk of recurrent stroke as pre-existing AF [13].

In the present study, we investigated whether specific brain areas are associated with newly detected AF after acute ischemic stroke. Specifically, we examined whether infarct locations differ between stroke patients with new AF, without AF and with AF already known before the stroke.

## Material and methods

### Patients and clinical data

Consecutive patients with evidence of acute cerebral infarction on diffusion-weighted magnetic resonance imaging (MRI) were included for analysis into the present observational study. All patients were aged >18 years and admitted to our acute stroke unit between 08/2009 and 09/2012. The National Institute of Health Stroke Scale (NIHSS) was applied to grade stroke severity. The medical history, basic demographic variables and cardiovascular risk factors were recorded using a standardized data collection form (S1 Data Collection Form; [14,15]). AF was classified as “known” if there was a documented history of AF. During the stay on the stroke unit patients underwent 12-lead admission ECG, 24h-Holter-ECG, and continuous ECG monitoring that included automated analysis software [16] dedicated to AF detection. New AF was diagnosed if it was found by at least one of these methods in the absence of a documented previous history of AF.

The manuscript was developed according to the STROBE guidelines for reporting observational studies [17]. The independent ethics committee of the Medical Faculty of the Heidelberg University approved collection of patient data after the patients or their legal representatives had given written informed consent for using their data (ethics statement S-290/2009).

### MRI protocol

Imaging was performed on 3 Tesla MRI units (Siemens TimTrio or Verio scanner) using a 12-channel phased-array head coil. The MRI protocol included diffusion-weighted imaging (DWI), which was performed by a single-shot spin-echo echo planar imaging (SE-EPI) sequence with three diffusion-encoding gradients along orthogonal axes at b = 1200 s/mm2 and a B0-acquisition with no diffusion weighting at a repetition time of 5300 ms, an echo time of 92 ms, effective echo spacing of 0.36 ms, a field of view of 250 by 250 mm, a matrix size of 130 by 130 pixels in-plane, a slice thickness of 5 mm, 28 slices, an interslice gap of 0.5 mm (10% of the slice thickness), using 4 averages and generating a voxelwise image of the apparent diffusion coefficient (ADC). Specifying the minimal possible effective echo spacing for the diffusion-weighted acquisitions minimized geometric EPI distortions due to magnetic field inhomogeneities.

### Lesion analysis

Ischemic stroke lesions were identified on diffusion-weighted imaging (DWI) by a board-certified neuroradiologist (AJB) [18]. At the individual level, preprocessing was performed using tools from the Oxford Centre for Functional Magnetic Resonance Imaging of the Brain’s Software Library (FMRIB Software Library FSL, version 5.0.9; http://www.fmrib.ox.ac.uk/fsl) [19] and consisted of (1) brain extraction by the Brain Extraction Tool (BET; part of FSL; [20]) and (2) eddy current as well as movement correction using eddy (part of FSL; [21]). Areas displaying diffusion restrictions in each of the three gradient directions and the ADC image were drawn manually on the transverse slices as regions of interest (ROIs) using FSLView (3.2.0, part of FSL). Each individual binary 3D infarct mask was then transformed to the structural standard space of the MNI152 template (part of FSL) by a full-affine registration matrix obtained from (3) co-registering the B0-image of the diffusion acquisition to the template using FMRIB’s Linear Image Registration Tool (FLIRT version 6.0; part of FSL; [22,23]) with 12 degrees of freedom, the inter-modal correlation-ratio cost function and nearest neighbor interpolation. (This approach was chosen because field maps or SE-EPIs with alternate phase encodings to estimate the susceptibility induced off-resonance field for distortion correction and high-resolution anatomical 3D-scans for nonlinear spatial registration to the MNI152 template were not available due to the time constraints of the clinical MRI protocol for acute strokes). Thereby, one three-dimensional, spatially standardized stroke lesion mask in MNI152 space was generated for each patient. These 3D-masks were merged across patients into a 4D-file by fslmerge (part of FSL) for further statistical analysis. Normalized total infarct volumes (in milliliters [ml]) were extracted in MNI152 standard space from these masks for each patient using fslstats (part of FSL), eliminating potentially confounding differences in brain (respectively head) size or total intracranial volume by the full-affine registration [24]. Left and right hemispheric masks were generated in MNI152 standard space around the x = 0 mm midplane using fslmaths (part of fsl), and normalized infarct volumes (in milliliters [ml]) were separated for the left and right hemisphere (by fslstats, like above) to estimate the impact of AF status on stroke size differences between both hemispheres across subjects (interaction effect). Voxelwise stroke lesion counts and the margins of 1% as well as 4% total empirical stroke probability were calculated for all patients and are displayed in “radiological convention” (left side of the brain shown on the right side of the figure) on six evenly spaced axial slices at z = -32, -12, 8, 28, 48 and 68 mm in MNI152 space. Similarly, empirical stroke probabilities were calculated separately on a voxel-by-voxel basis for patients with new AF, known AF (both separately and combined) and for those without AF. These empirical stroke probabilities are displayed in percent on the same axial slices in MNI152 space.

### Statistical analysis

#### Statistical image analysis with Bayesian Spatial Generalised Linear Mixed Modeling for voxel-based lesion–symptom mapping

To examine relationships between ischemic tissue damage and the occurrence of AF on a voxel-by-voxel basis, we performed voxel-based lesion-symptom mapping (VLSM) [25]. Binary stroke maps in 4D (0 = no stroke vs. 1 = stroke lesion present, coded for each voxel in 3D template space and for each patient in the fourth dimension) were analyzed using the Bayesian Spatial Generalised Linear Mixed Model (BSGLMM) of a spatial probit regression approach with spatially varying coefficients [26]. Accounting for the discrete nature of the data, BSGLMM (freely available at http://warwick.ac.uk/tenichols/BSGLMM) is similar to a logistic regression but with spatial regularization. Note that BSGLMM models voxelwise lesion data as the response while VLSM conventionally models it as the predictor. Given that there is no doubt that AF can indeed cause strokes, it is more adequate to model ischemic lesions as responses and clinical status (new AF, known AF and no AF) as potential predictors than vice versa.

Since there were differences in infarct volume between the diagnostic groups (c.f. Results), we first examined which brain areas were associated with increasing stroke size using power transformed, cubic root normalized total infarct volume (crIV; in millimeters [mm]) as a BSGLMM predictor (note that cubic root stroke volume is a “natural choice” because the number of voxels involved in a stroke increases linearly with it). Then, the following voxelwise comparisons of infarct load were performed using BSGLMM: (I) new AF vs. no AF and (II) new AF vs. known AF. All analyses used age and gender as nuisance covariates. The following three analyses were carried out to examine differences of infarct locations for the aforementioned comparisons: 1) base model (with age and gender as nuisance covariates), 2) with the additional explanatory variable of crIV (see above) as a covariate, 3) analysis of a range-matched subsample in which crIV did not differ between the diagnostic groups (new AF, known AF and no AF). In the third analysis crIV was not explicitly modeled because the matching eliminated previous crIV differences between diagnostic groups (c.f. Results), and hence there should be no significant confounding effect attributable to total infarct volume for the between-group comparisons.

For statistical inference, the standardized BSGLMM coefficients of the comparisons were transformed from z- into p-values using fslmaths (part of FSL). Multiple comparison correction was performed by thresholding the results within the margins of a mask of 1% total empirical stroke lesion probability at a False-Discovery Rate (FDR) [27–29] of q = 0.01 using fdr (part of FSL) so that the expected proportion of false positives among suprathreshold voxels did not exceed 1%. Note that setting the inclusion threshold of total empirical stroke lesion probability above 1% would have favored right hemispheric detections per se, with a particular bias towards the right insula.

FDR-corrected results were rendered on glass brain projections [generated using the MNI152 brain edge image and fslmaths (part of FSL)] and the MNI152 template (using Renderstats, part of FSL). Colour-coded two-tailed FDR-corrected results at q = 0.01 are displayed in “radiological convention” on glass brain projections and six axial evenly spaced slices (at z = -32, -12, 8, 28, 48 and 68 mm) in MNI152 space. Contiguous voxels were tabulated using cluster and atlasquery (using the Havard-Oxford Cortical and Subcortical Structural Atlas for reference; both part of FSL) for all clusters exceeding 25 voxels. Coordinates for the center-of-gravity (COG) are given in MNI152 template voxel space.

#### Other statistical analyses

Descriptive clinical data are presented in relative frequencies, ordinal and continuous data as medians and interquartile ranges (IQR). Differences of patient characteristics were examined by univariate nonparametric tests. A two-sided p value of ≤0.05 was considered significant. Data analysis was performed using the Statistical Package for the Social Sciences (SPSS 19.0) and R Project for statistical computing (version 3.3.2) [30].

## Results

### Patients and basic demographic findings

**Fig 1:**
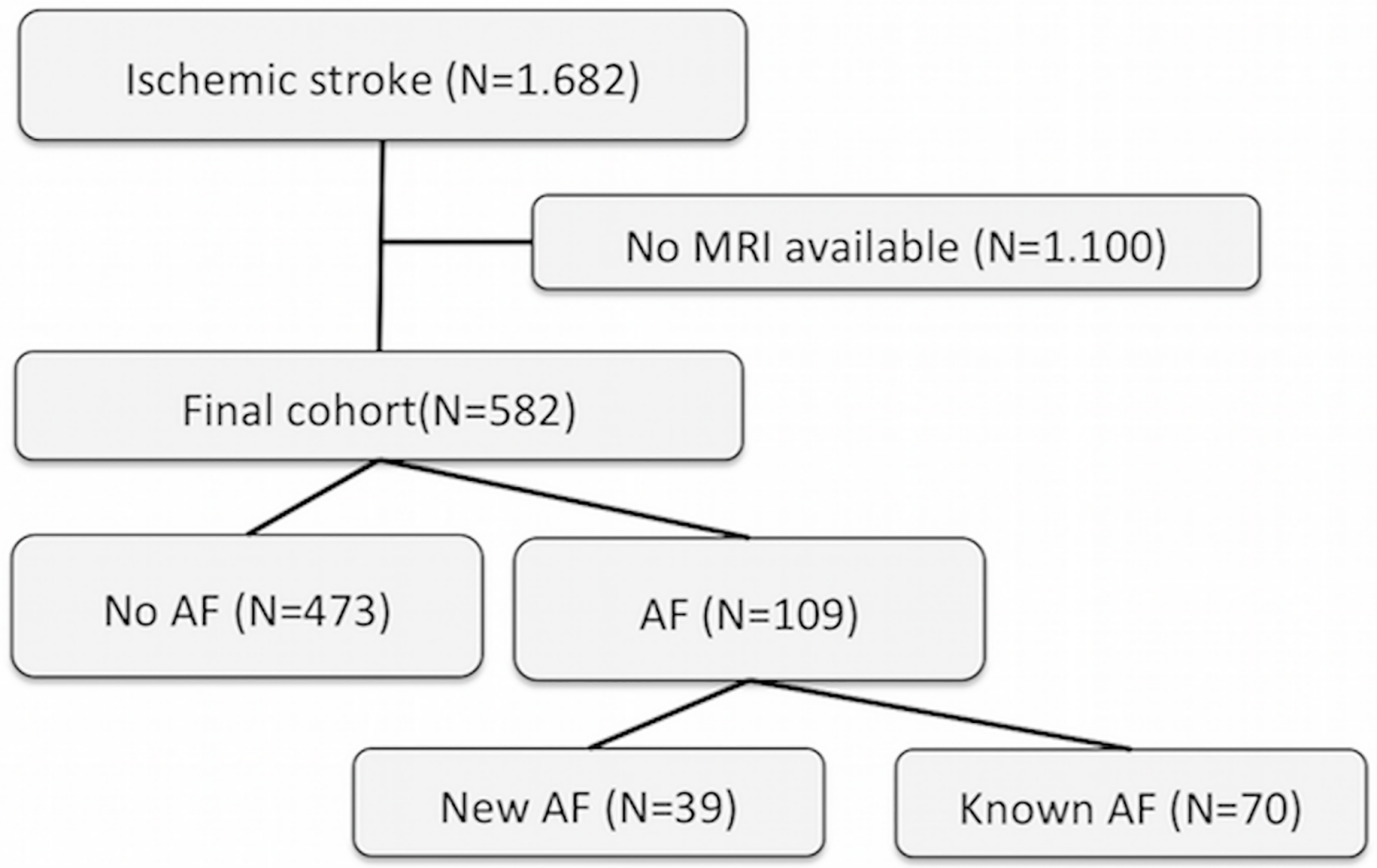
Composition of the analyzed cohort of patients. (AF: atrial fibrillation, MRI: magnetic resonance imaging, N: number).

The analyzed cohort comprised 582 patients (c.f. Fig 1). Median age was 68 years (IQR 56–75), 63% were male (Table 1). Brain DWI was conducted 28 h 29 min (median) after the onset of stroke symptoms (IQR 7:24–70:59).

**Table 1:** Clinical characteristics of included patients. (AF: atrial fibrillation; NIHSSS: National Institute of Health Stroke Scale Score; IQR: Interquartile range; n: number; SD: standard deviation).

	No AF (n = 473)	New AF (N = 39)	p	New AF (N = 39)	Known AF (n = 70)	p
**Male sex; N (%)**	309 (65.3)	13 (33.3)	<0.001	13 (33.3)	46 (65.7)	<0.001
**Age; years [(median (IQR)]**	65 (53–73)	75 (72–81)	<0.001	75 (72–81)	77 (70–81)	0.400
**NIHSSS; [median (IQR)]**	3 (2–6)	8 (2–17)	<0.001	8 (2–17)	5 (2–13)	0.253
**Arterial hypertension; N (%)**	356 (75.3)	36 (92.3)	0.026	36 (92.3)	61 (87.1)	0.532
**Diabetes; N (%)**	105 (22.2)	6 (15.4)	0.418	6 (15.4)	23 (32.9)	0.070
**Prior stroke/TIA; N (%)**	71 (15.0)	7 (17.9)	0.037	7 (17.9)	22 (31.4)	0.175
**Coronary heart disease; N (%)**	52 (11.0)	9 (23.1)	0.079	9 (23.1)	23 (32.9)	0.381
**Oral anticoagulation at admission**	12 (2.5)	0 (0)	0.613	0 (0)	31 (44.3)	<0.001
**Symptom-onset to MR-imaging [(hours:min, median (IQR)]**	29:35 (08:45–71:19)	11:19 (4:42–38:11)	0.010	11:19 (4:42–38:11)	17:30 (05:16–74:46)	0.269
**Normalized infarct volume; ml (mean± SD)**	15.1 ± 35.1	41.2 ± 52.0	0.001	41.2 ± 52.0	23.3 ± 41.0	0.171

Atrial fibrillation was present in 109 patients (18.7%). In 36% of these (39/109), AF was detected for the first time on the stroke unit (new AF) and the median time-interval between hospital admission and AF detection in these patients was 9h 30min (IQR 3:04–34:00). In 64% (70/109) AF had been diagnosed already before the stroke (known AF). Oral anticoagulants were not used at admission in any of the patients with new AF (0%) but in 31/70 patients with known AF (44%; p<0.001).

Overall, 17% of the entire sample of stroke patients (n = 100/582) had previous strokes in their medical history (Table 1). In 29 of these, AF was confirmed by discharge (known AF: 22, new AF: 7). Median CHA_2_DS_2_-VAsc-Score in these 29 AF patients was 6 (IQR 5–7) and did not differ between known AF (median CHA_2_DS_2_-VAsc-Score 6, IQR 5–7) and new AF patients (median CHA_2_DS_2_-VAsc-Score 6, IQR 5–6; p = 0.39).

In the overall cohort of patients with AF (109), symptomatic carotid artery stenosis was present in eight cases (7.3%) while it was observed in 89/473 patients without AF (18.8%; p = 0.01). No difference was detected with regard to the presence of symptomatic carotid artery stenosis between the AF groups: Here, 6/70 patients (8.6%) with known AF and 2/39 (5.1%) with new AF were affected (p = 0.71).

### Stroke lesion count map, empirical stroke probabilities and infarct volumes

**Fig 2:**
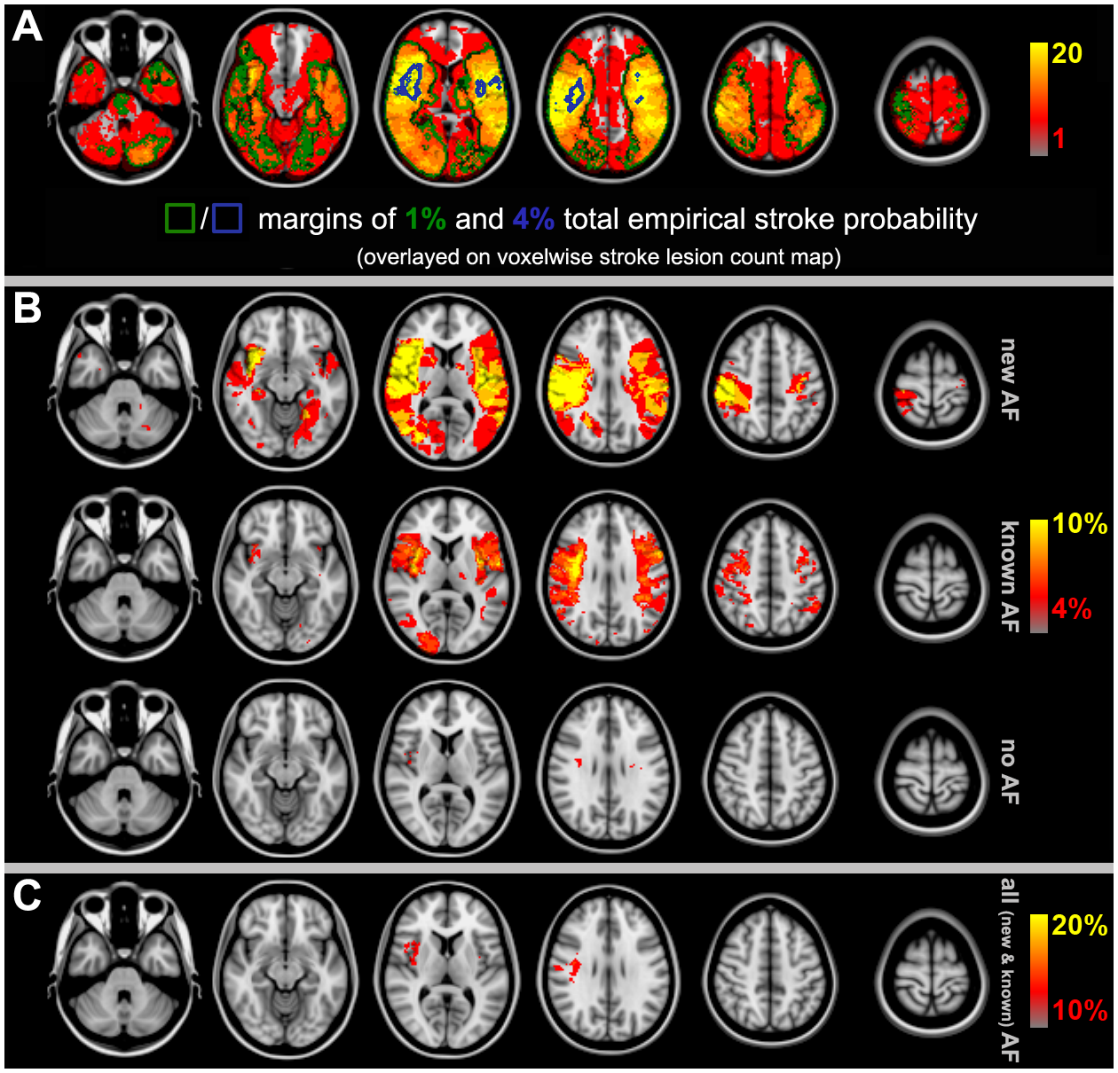
Voxelwise stroke lesion counts for the whole cohort and the diagnostic subgroups of different AF status. (A): Voxelwise stroke lesion counts and the margins of 1% and 4% total empirical stroke probability for the entire cohort of 582 acute ischemic stroke patients. Note the accumulation of ischemic lesions in the right insula, as illustrated by the 4% stroke probability margin. (Red-to-yellow: stroke lesion counts scaled between 1 to 20 lesions per voxel; green: margin of 1% total empirical stroke probability; blue: margin of 4% total empirical stroke probability). (B): Voxelwise empirical stroke probabilities for patients with new (top), known (middle) and without (bottom) AF. Smaller infarct volumes in patients without AF (cf. Fig 3) lead to less overlap of the ischemic lesions across subjects and thus much fewer voxels above the 4% empirical stroke probability threshold (set to display for illustration) while for the larger strokes of new and known AF ischemic lesions accumulate in both middle cerebral artery territories and centrally around the insula, in particular. (C): Voxelwise empirical stroke probabilities (thresholded at 10%) reveal that AF (new and known combined) leads to an accumulation of stroke lesions in the right MCA territory and insula, in particular. (AF: atrial fibrillation; MCA: middle cerebral artery).

The voxelwise stroke lesion count map for the entire cohort is shown in Fig 2A. Overall, lesions in the central MCA territory, particularly the right insula, were more prevalent than lesions in other locations. Fig 2B displays empirical stroke probabilities voxelwise and separately for patients with new AF, with known AF and without AF while Fig 2C shows the combined empirical stroke probability for patients with new and known AF.

Normalized (i.e., adjusted for brain size) infarct volumes in our patients ranged between 0.016 and 270.792 ml and were strongly right-skewed to higher values (median = 2.3 ml; mean = 17.9 ml). To obtain a more symmetric distribution, normalized infarct volumes were power transformed into cubic-root infarct volumes (crIV), which resulted in a less skewed distribution (median = 13 mm, mean = 18 mm). Therefore, crIV was (in analysis 2, c.f. section Methods) used as an explanatory covariate for the subsequent voxelwise stroke mapping in BSGLMM to examine differences of infarct locations between the diagnostic groups of different AF status (new AF, known AF, no AF).

**Fig 3:**
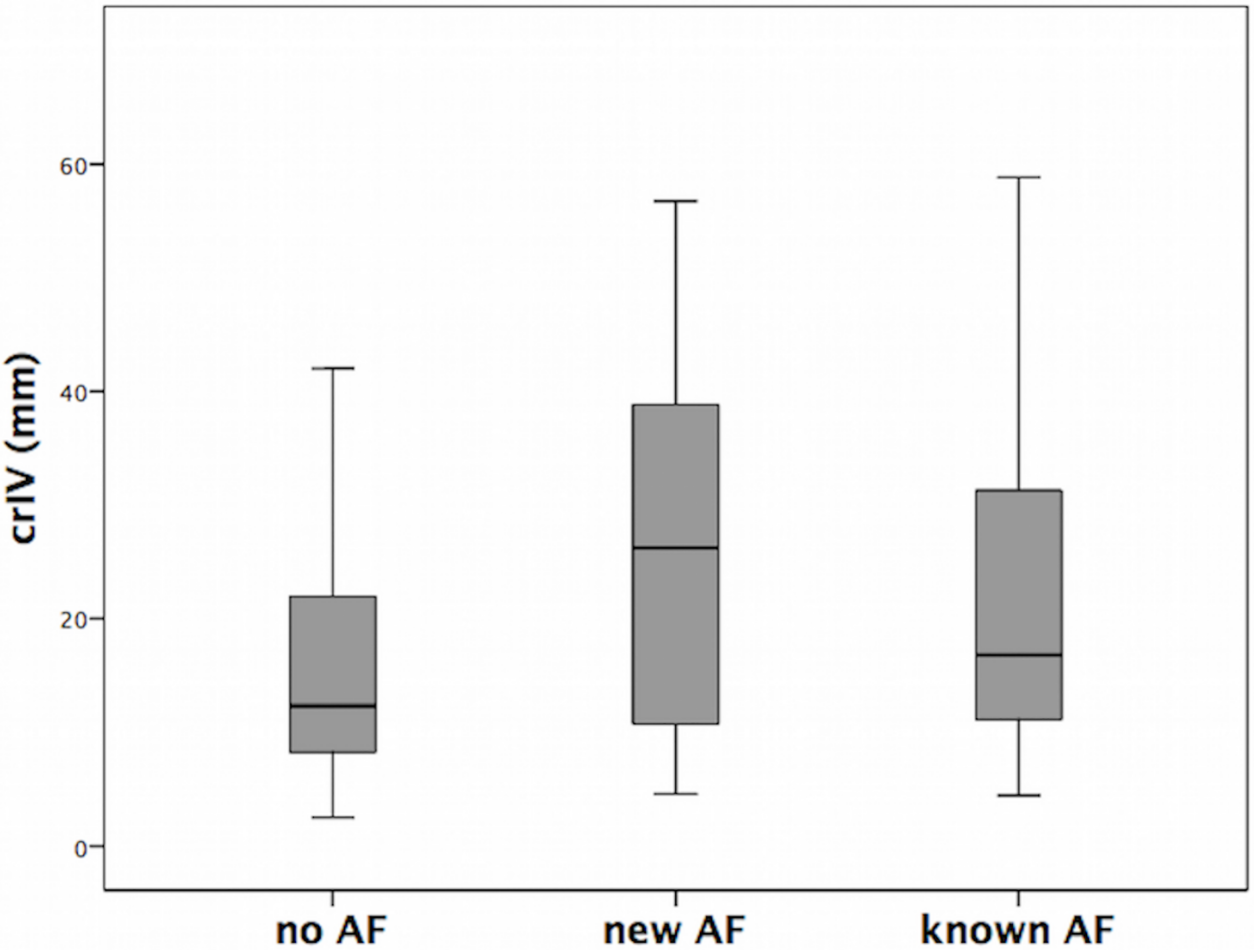
Cubic root total infarct volume by AF status (no, new and known AF).

Cubic root total infarct volume (crIV) was, adjusted for the impact of age and gender, significantly influenced by AF status (no, new or known AF; ANCOVA F = 22.50; p<0.001; cf. Fig 3). Normalized infarct volume was higher for patients with AF (mean 29.7 ± 45.8 ml) than for those without AF (mean 15.1 ± 35.1 ml; p<0.001). Patients with new AF (mean 41.2 ± 52.0 ml) had, on average, larger infarcts than patients with known AF (mean 23.3 ± 41.0 ml) but the difference between them did not reach statistical significance (p = 0.17). Normalized for brain size, infarct volume did not differ between known AF patients with and without oral anticoagulation at admission (22.9 ± 40.0 ml and 23.7 ± 42.2 ml respectively; p = 0.69).

Aside from presenting with larger strokes, infarcts of patients with AF from our sample accumulated in the right more than in the left MCA territory centered to the insula (Fig 2C). Right hemispheric strokes in AF were, on average, larger than in the left hemisphere, both in new and, slightly less, in known AF of our cohort (new AF: 24.1 ± 47.8 ml vs. 17.0 ± 35.4 ml; known AF: 21.8 ± 42.4 ml vs. 18.1 ± 33.9 ml). Strokes in patients without AF were not only significantly smaller but did also not reveal such interhemispheric difference (7.5 ± 23.3 ml vs. 7.6 ± 26.3 ml). Adjusted for age and gender, a statistically significant interaction effect between AF status and hemispheric involvement on the normalized number of stroke voxels (as given by crIV) was observed (p = 0.02).

To eliminate the confounding impact of stroke size on group comparisons between AF states we included crIV as an additional explanatory variable (in analysis 2; c.f. section Methods). Additionally, we sampled all cases within the smallest range of the crIV leaving n = 20 cases with new AF. This crIV range corresponded to normalized infarct volumes of 0.096 to 18.064 ml. In the related subsample (457 patients: 20 with new AF, 48 with known AF and 389 without AF) crIV was no longer influenced by AF status (ANCOVA F = 2.20, p = 0.14). Hence, these cases were used for the crIV-matched subsample comparisons of voxelwise BSGLMM analysis 3 (c.f. section Methods).

### Association of stroke size and infarct location

**Fig 4:**
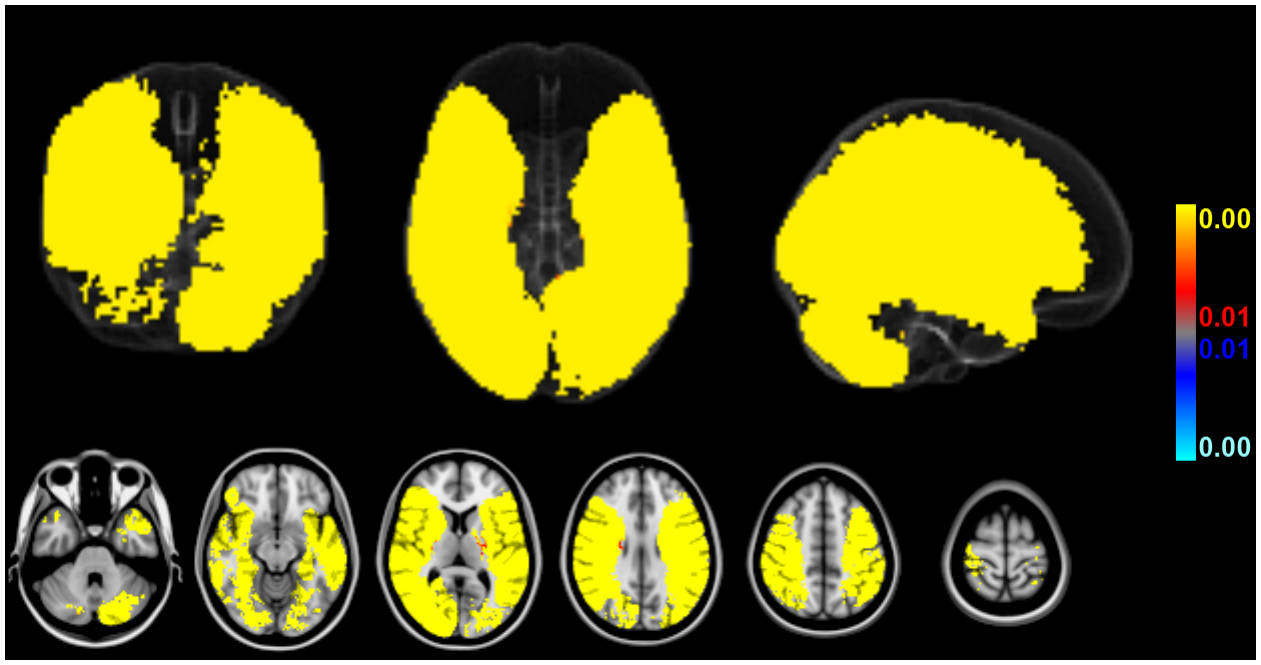
Association of stroke size and voxelwise ischemic involvement across 582 patients. Larger strokes were associated with more frequent ischemic lesions in the MCA territory of both hemispheres and parts of the posterior circulation while smaller strokes were not associated with more frequent ischemic insults of any brain area. (Red-to-yellow: areas significantly associated with higher crIV, blue-to-lightblue: areas significantly associated with lower crIV across all n = 582 patients; thresholded at FDR-corrected q = 0.01; age and gender included as covariates of no interest, AF status unmodeled).

Adjusted for age and gender (as well as brain size but irrespective of AF status), increasing stroke size (as modeled by crIV) was associated with significantly more ischemic lesions primarily in the territory of the middle cerebral artery (MCA) of both hemispheres but also parts of the posterior circulation across the entire sample of patients. Decreasing stroke size (as modeled by crIV) was, on the other hand, not associated with significantly more ischemic insults to any consistent brain area (Fig 4).

Thus, more frequent infarcts within the brain areas detected in Fig 4 may potentially not result from “true” group differences of AF status (new, known or no AF) but instead from the confounding effect of larger stroke sizes (if crIV is not matched or included in the analysis). Note that the brain areas in which the likelihood of more frequent stroke lesions increases linearly with cubic root stroke volume (at FDR-corrected q = 0.01) cover almost the entire inclusion mask of 1% total empirical stroke lesion probability (98.9% coverage; cf. Fig 4 with Fig 2A). Only voxelwise involvement of pons and both thalami was not predicted by progressively increasing stroke size. In fact, standardized BSGLMM coefficients were negative in these subcortical regions (not shown). Also note that sparing of the territory of the anterior cerebral artery (ACA) of both hemispheres does not reflect a true-negative detection but a priori exclusion from the BSGLMM analysis (because strokes in this territory did not exceed the minimum voxelwise inclusion probability of 1%). Unmasked standardized BSGLMM coefficients attained high values also in the ACA territory, and using a more liberal inclusion mask (below a voxelwise total empirical stroke probability of 1%) would have yielded positive detections in the ACA territory as well (not shown). Given that such extended inclusion would, however, correspond to 5 or less ischemic lesions per voxel across the entire sample, we considered results obtained at more liberal inclusion masking to be of questionable validity.

### Differences of infarct locations: New AF versus no AF

**Fig 5:**
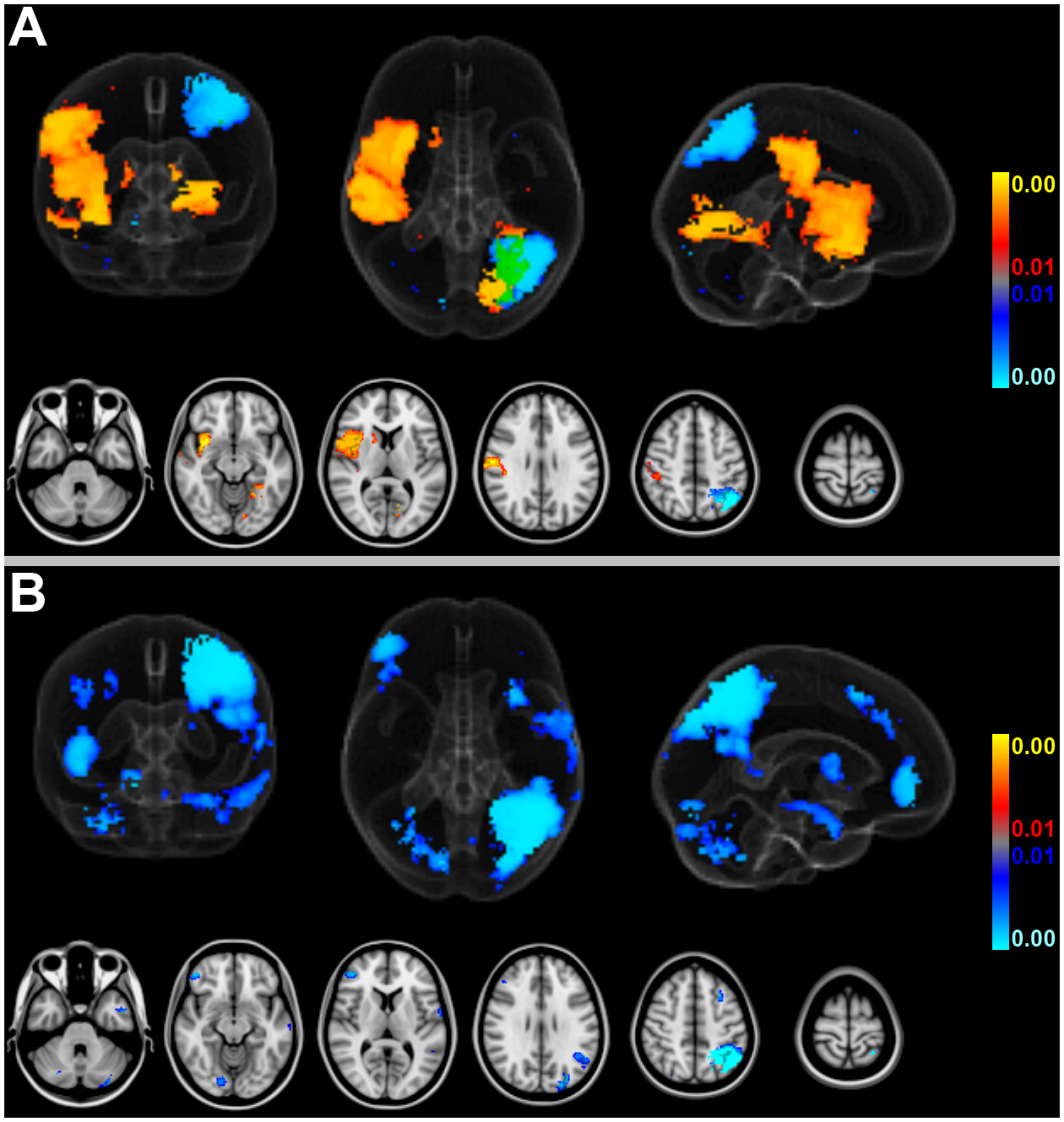
Differences of infarct locations: New AF versus no AF. (A): New AF was, compared to patients without AF, associated with ischemic lesions in a) the right (peri-) insular cortex and the caput of the right caudate nucleus and b) in the left PCA territory. Patients without AF had significantly more often strokes in the left parietal lobe than patients with new AF. (Red-to-yellow: areas significantly more often associated with strokes in patients with new AF vs. without AF, blue-to-lightblue: vice versa and green: overlap projections; thresholded at FDR-corrected q = 0.01; age and gender were included as covariates of no interest, with infarct volume unaccounted for in this analysis). (B): Including infarct volume into the analysis revealed that no specific brain region was associated with significantly more stroke lesions in patients with new AF compared to patients without AF. Vice versa, patients without AF still had more often left parietal involvement compared to patients with new AF. (Red-to-yellow: areas significantly more often associated with strokes in patients with new AF vs. without AF [none], blue-to-lightblue: vice versa; FDR-corrected q = 0.01; with removal of the confounding effect of cubic root infarct volume normalized for brain size, age and gender).

In the overall cohort, and controlled for age and gender, new AF was associated with more frequent lesions in the right (peri-) insular cortex and the right caudate nucleus (i.e., in central territories of the right MCA) as well as parts of the left posterior cerebral artery (PCA) territory compared to patients without AF. Vice versa, patients without AF had significantly more often ischemic lesions particularly in the left parietal lobe compared to patients with new AF (Fig 5A, Table 2).

**Table 2:** Tabulated stroke areas that significantly differed in the comparisons between diagnostic groups (without correcting for infarct volume, BSGLMM-VLSM, FDR-corrected q = 0.01 within 1% of total empirical stroke probabilities).

**New AF compared to no AF**						
**Number of Voxels**	**lowest FDR p-value**	**z-value@COG**	**COG X (vox)**	**COG Y (vox)**	**COG Z (vox)**	**Location**
4165	0.002	5.17	26	66	28	right (peri-) insular cortex
568	0.002	5.08	54	28	36	left lingual gyrus
112	0.003	3.92	13	60	27	right middle temporal gyrus
38	0.004	3.68	36	72	42	right caudate
29	0.002	4.14	51	27	40	left intracalcarine cortex
**No AF compared to new AF**						
**Number of Voxels**	**lowest FDR p-value**	**z-value@COG**	**COG X (vox)**	**COG Y (vox)**	**COG Z (vox)**	Location
1674	0.001	3.55	57	38	58	left superior parietal lobule
**New AF compared to known AF**						
**Number of Voxels**	**lowest FDR p-value**	**z-value@COG**	**COG X (vox)**	**COG Y (vox)**	**COG Z (vox)**	Location
2783	0.000	5.26	13	60	28	right middle temporal gyrus
25	0.004	3.71	14	55	56	right parietal lobe (postcentral gyrus)

Note that all voxels detected in these comparisons (Fig 5A) are within infarct locations revealing a significant crIV-dependent effect (Fig 4), i.e. these detections are potentially confounded by stroke size. After including crIV as a confounder into the analysis, no specific brain region was associated with new AF compared to patients without AF anymore, indicating that the aforementioned finding depended on stroke size. Patients without AF still exhibited more frequent left parietal involvement compared to patients with new AF (Fig 5B).

In the crIV-matched subsample analysis, no significant difference of infarct location between patients with new AF and patients without AF or vice versa was observed.

### Differences of infarct locations: New AF versus known AF

**Fig 6:**
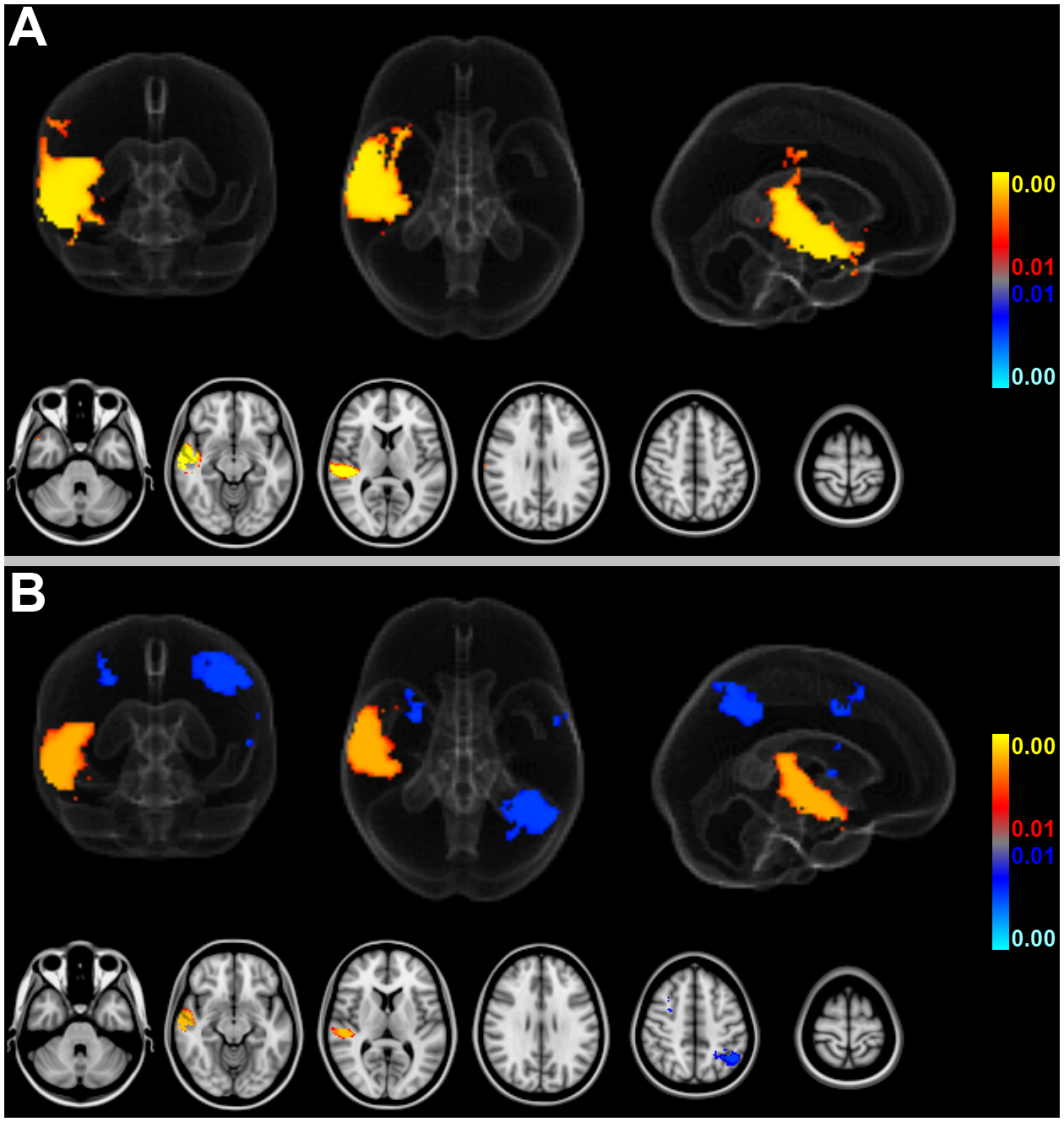
Differences of infarct locations: New AF versus known AF. (A): New AF was associated with more strokes in the central territory of the right MCA than known AF. The reverse comparison of known AF versus new AF did not yield a significant difference. (Red-to-yellow: areas significantly more often associated with strokes in new AF vs. known AF patients; blue-to-lightblue: vice versa; FDR-corrected q = 0.01; age and gender were included as confounds, with infarct volume unaccounted for in this analysis). (B): New AF was also more often associated with right central MCA-strokes compared to known AF after including infarct volume as a confound. Here, the left parietal lobe was significantly less often affected in new AF compared to known AF. (Red-to-yellow: areas where new AF was associated with significantly more strokes than in patients with known AF; blue-to-lightblue: vice versa; FDR-corrected q = 0.01; with removal of the confounding effect of cubic root infarct volume, age and gender).

In patients with new AF, and controlled for age and gender, lesions in central locations within the right MCA territory beyond the insula were significantly more frequently detected than in patients with known AF (Fig 6A and Table 2). No regions were associated with more lesions in patients with known AF compared to patients with new AF.

Note that all voxels detected in this comparison (Fig 6A) fall again into infarct locations revealing a significant crIV-effect (Fig 4), i.e. these detections are also potentially confounded by stroke size. After including crIV along with age and gender as a further nuisance covariate, new AF was still more often associated with strokes in the central territory of the right MCA. Vice versa, left parietal involvement was then less often detected in patients with known compared to new AF (Fig 6B).

The analysis of lesions in the crIV-matched subsample revealed no significant differences of infarct locations between patients with new AF compared to known AF or vice versa.

Overlap of stroke lesions in MNI152 standard space between group comparisons of patients with new AF versus no AF (Fig 5) and group comparisons of patients with new AF versus known AF (Fig 6) was in total only 312 voxels (i.e., 6%).

## Discussion

Our investigation highlights the need to account for stroke size in searching for infarcted brain areas that may trigger cardiac arrhythmias. AF was confirmed to be associated with larger strokes compared to patients without AF. The major new results of our study are i) that no distinct brain area can at present be specifically implied to trigger AF when confounding stroke size is adjusted for and ii) that left parietal involvement is significantly less likely in AF newly detected after an ischemic stroke. Irrespective of the question whether neurogenic AF exists, such left parietal lobe lesions are, of all potential brain areas, most certainly unrelated to new onsets of AF.

Imbalances of central autonomic control due to ischemic brain lesions and resulting alterations of brain-heart interactions have been suggested to provoke cardiac arrhythmias after acute stroke [2,3,5,7,9,11,31,32]. It is, however, a matter of an ongoing debate whether newly diagnosed AF represents the consequence of specific acute brain lesions or is simply a previously silent and undetected cause of the infarct [2,7,33–38]. In prior studies, right insular ischemic infarcts have been associated with electrocardiographic abnormalities including AF [3,5,9]. Only one of these studies used voxelwise stroke lesion image analyses but did not specifically focus on patients with newly diagnosed AF and instead included various types of cardiac arrhythmias [9]. Another recent study reported that newly detected AF during in-hospital ECG monitoring was associated with insular cortex involvement in ischemic stroke [8]. Here, only the frequency of insular stroke involvement was recorded and no voxelwise analysis was performed [8]. Stroke size was not accounted for in any of these studies. Therefore, it has remained unresolved whether insular insults reflect a true cause of AF newly detected after stroke or just a frequent destination of cardiogenic emboli.

In our patients, new AF was associated with infarcts in the right MCA territory, including (but not limited to) peri-insular regions (Fig 5A). However, right insular lesions were more prevalent than strokes in other locations in the entire cohort of included patients suggesting that this brain area per se represents a frequent target of ischemia in stroke patients (Fig 2A). Cardio-embolic infarcts due to AF are usually larger compared to atherothrombotic infarcts [12,39], presumably because cardiac emboli are larger than those from other sources. Correspondingly, infarct volumes differed depending on presence or absence of AF in our study, and infarcts in patients with AF were considerably larger compared to patients without AF (Fig 3). Larger strokes, in turn, increased territorial cortical involvement but spared the brainstem and both thalami (Fig 4). This finding makes sense given that brainstem and thalamic infarcts are often lacunar in nature. In fact, within the inclusion mask only the voxelwise involvement of pons and both thalami was itself not linearly predicted by cubic root total stroke volume (normalized for brain size) which illustrates an important difference between cortical territorial versus subcortical lacunar infarctions (cf. Fig 4 with green margins in Fig 2A). Furthermore, our data reveal that AF is associated not only with larger infarcts but, on average, with stroke lesions mainly in the central MCA territory of both hemispheres (Fig 2B). These affected the right more often than the left insula (Fig 2C). Taken together our findings confirm that these brain areas represent frequent targets of cardio-embolism, rather than a cause of newly diagnosed AF. Larger strokes affecting primarily the MCA territory are more likely to overlap, leading to higher empirical infarct probabilities within these areas for patients with AF (both new and known) compared to patients without AF (Fig 2B). Including infarct volume as a confounding factor into our voxelwise approach then revealed no evidence for any specific brain area being more often affected by strokes in patients with new AF compared to patients without AF (Fig 5B). This result was further corroborated by a subsample analysis of matched infarct volumes. It suggests that the differences of infarct locations between patients with new AF compared to patients without AF, which were only present in areas whose involvement simultaneously depended on infarct size, primarily result from larger infarct sizes in patients with new AF when infarct volume is not controlled for. Yet, left (but not right) parietal involvement was consistently less likely in AF newly detected after stroke. The most plausible explanation for the last-mentioned finding is, in our opinion, twofold: First, smaller emboli are more likely to end up in this territorial border zone and particularly smaller strokes of other than primary cardiogenic origin in the group without AF are more likely to involve parietal areas (e.g., hemodynamic or small thromboembolic strokes). Second, such parietal detections are influenced by the interaction between diagnostic groups and hemispheric involvement on stroke size, i.e. the fact that especially new AF was not only associated with larger strokes in general but also with progressively larger right than left hemispheric strokes specifically. In patients without AF such interhemispheric difference was not present. Supposing that larger strokes extend more peripherally, right superior parietal lobule involvement can be expected to remain below statistical significance without adjusting for lesion volume while the power to detect such difference in the right hemisphere after adjusting for lesion volume may have been too low. These assumptions are further supported by the observation that the standardized BSGLMM coefficients of right superior parietal lobule involvement were as well negative (-1.60 adjusted for crIV at [27, 38, 58] MNI152 voxel coordinates on the right, as opposed to -4.10 at [57, 38, 58] on the left for the new AF > no AF comparison). In other words, the BSGLMM model fit indicates that, on average, right parietal involvement is also less frequent for new onset AF but the effect was not statistically significant with or without adjustment for lesion volume.

We also compared lesion locations between patients with new AF and AF already known before the stroke. We hypothesized that—in case that newly detected AF had been present already before the stroke and hence caused cardiogenic cerebral embolism—patients with new AF would have similar infarct locations as patients with known AF. Instead, patients with newly diagnosed AF suffered more often from infarcts in right superior temporal lobe compared to patients with known AF, both according to the base model and after including crIV as an additional explanatory variable, but not in the matched crIV subsample analysis (Fig 6). The reason for this discrepancy remains speculative, but the right superior temporal lobe has not been previously suggested to be involved in cardiac control or in triggering poststroke AF. The detections by the base model and the model including crIV as a covariate for the new AF vs. known AF comparison may, for example, reflect a nonlinear dependence on crIV. Alternatively, a reduced power of the crIV-matched group comparisons (performed to eliminate an influence of crIV by AF status) may have contributed to the discrepant findings (1^st^ analysis n = 582; 2^nd^ analysis n = 457). Nonetheless, these areas differed clearly from those that were identified in the comparison between patients with new AF and those without AF when stroke size was not accounted for.

Our findings partially correspond to a smaller study (with a total of 150 included patients, using VLSM of strokes detected by CT or MRI) that compared associations of left and right hemispheric lesions to various poststroke cardiac arrhythmias including AF [9]. In that study, an association between the occurrence of cardiac arrhythmias and lesions in the right MCA territory including the insula was found but the association was, in fact, no longer significant when infarct size was included as a covariate. Median stroke volume was, even in this smaller sample, larger for patients with than without arrhythmia. This supports our notion that right insular involvement primarily results from larger infarcts which are themselves associated with AF. Moreover, the study did not discriminate between patients with new AF versus patients with AF known prior to the recent stroke [9], and the analysis was probably further confounded by a voxel inclusion bias due to setting the threshold of total empirical stroke lesion probability to 5% which, according to our data, strongly favors right insular detections (cf. blue margins in Fig 2A). Accumulation of stroke lesions in the right MCA territory and insula, in particular, from AF (new and known; Fig 2C) may simply result from the vascular anatomy given that the brachiocephalic trunk is, as the first and largest aortic branch, prone to catch cardiac emboli.

Although our findings argue against the concept that particular infarct locations trigger the development of AF after a stroke, we cannot rule out that other neurogenic mechanisms may participate in the development of new AF after brain ischemia. It is conceivable that “neurogenic” AF after ischemic stroke is not triggered by circumscribed lesions of cortical or subcortical brain areas but by disturbances of more distributed cerebral networks controlling autonomic and atrial function. Lesions of such functional networks may remain ambiguous to VLSM and instead require lesion-network mapping (LNM) to be detected [40]. Instead of being related to regionally specific stroke effects, it could also be just the infarct size per se that matters so that AF may result from autonomic imbalances after large strokes irrespective of their location. On the other hand, stroke patients with new AF and with known AF share common cardiovascular risk factors, have similar echocardiographic findings, and suffer from equally severe strokes [15], supporting the notion that pre-existing heart disease represents the most likely cause of newly detected AF after stroke. A recent study compared the prevalence of new AF in patients suffering from ischemic stroke and TIA with patients suffering from intracranial hemorrhages (ICH) [41]. Given that new AF was very rare in the ICH group, the authors concluded that intracranial lesions rarely provoke new AF but that stroke is often the first manifestation of new AF in younger patients with no history of cardiovascular disease [41]. These data further support the assumption that AF represents the cause rather than a consequence of acute ischemic stroke and TIA. However, evidence from that study still remained somewhat inconclusive because i) no voxelwise analysis was performed and ii), though inclusion of an ICH group clearly represents a strength, neuronal damage differs between ischemic stroke and intracranial hemorrhages (with ischemic strokes being primarily hypoxic and intra-axial hemorrhages primarily space-occupying lesions).

Our study has strengths and limitations. Instead of “conventional” VLSM we used BSGLMM and modeled ischemic lesions as voxelwise responses and clinical status of AF as potential predictors in a well-defined group of stroke patients with new AF, known AF and without AF, using a base model and two further analyses controlled and matched for stroke volume size. Excluding all AF patients revealing topographic stroke lesion patterns associated with potentially alternate stroke etiologies (i.e., lacunar and hemodynamic strokes) did not alter our results. Moreover, we analyzed a comparatively large cohort and focused specifically on AF rather than on a heterogeneous group of arrhythmias. Although continuous ECG monitoring was performed in all patients during their stay at the stroke unit, we cannot exclude that a few patients categorized as having no AF may have had undetected AF and the total number of new AF patients is limited. It is also conceivable that patients with known AF may have had prior stroke lesions that had preceded (and thus potentially triggered) AF and we did not investigate any inflammatory responses. Furthermore, different etiologies can result in different infarct patterns on imaging but the cause of a stroke cannot be reliably deduced from the stroke pattern alone (i.e., lacunar strokes can be atheromatous, thrombotic or embolic in nature). A lack of further details concerning the classification of acute ischemic stroke subtypes (i.e., according to TOAST criteria) may limit our results. Inclusion of patients with a history of stroke could also represent a confounder but their exclusion (by confound modeling; not shown) did not alter our main results or their interpretation. Because less than half of the eligible consecutive stroke patients received an MRI for stroke confirmation, a selection bias and insufficient statistical power to detect potential differences between new AF and no AF controlled for stroke size cannot be ruled out. Future studies should therefore pool their data across dedicated stroke centers in order to further increase the power and consider using lesion-network mapping approaches to test the impact of stroke lesions, controlled for stroke size, on functional networks that may regulate autonomic cardiac control and hence might, irrespective of our findings, cause AF.

## Conclusions

Strokes in patients with AF tend to be larger than in patients without AF and are, possibly due to the underlying vascular anatomy, biased towards right insular involvement. While left parietal infarctions were less prevalent in patients with AF newly detected after stroke, we did not find evidence that specific brain areas are, controlled for infarct size, consistently associated with new AF after ischemic stroke. This challenges the concept that a relevant proportion of new atrial fibrillation after a stroke is triggered by circumscribed ischemic brain lesions.

## Supporting information

S1 Data Collection Form (PDF)
