# Supplementary material for: Voxelwise distribution of acute ischemic stroke lesions in patients with newly diagnosed atrial fibrillation: Trigger of arrhythmia or only target of embolism?
Source: PLoS One. 2017 May 24;12(5):e0177474. doi: 10.1371/journal.pone.0177474 (PMC5443524; doi:10.1371/journal.pone.0177474)
Supplement: S1 Data Collection Form — (PDF) [file pone.0177474.s001.pdf]

Description of variables

column	variable	extra (if not given: 1=y 0=n)
1	Gender	-1=male,1=female
2	Age	
3	AF at discharge	
4	Known_AF	
6	New_AF	
8	Time of stroke onset	
9	NIHSS_on_admission	
10	Arterial hypertension	
11	Congestive heart failure	
12	Diabetes	
13	Peripheral Arterial Occlusive Disease	
14	Prior stroke	
15	CHADS2DS2-VASC score	
16	Symptom_to_NMR time	[min] (-1 = no info)
17	Vitamin_K_antagonist_on_admission	
18	NOAC_on_admission	
19	Antiplatelets_on_admission	
20	Microbleeds (BOMBS-Score)	
21	White_mater_lesions (Scheltens score)	
22	Cholesterol_on_admission	[mg/dl]
23	Ldl_on_admission	[mg/dl]
24	Symptomatic carotid artery stenosis	
25	Echocardiography	
26	Echo_Ao	[mm]
27	Echo_LA	[mm]
28	Echo_Septum_size	[mm]
29	Echo_posterior_wall_size	[mm]
30	Echo_LV_ED	[mm]
31	Echo_LV_ES	[mm]
32	Echo_RV	[mm]
33	Echo_VCI	[mm]
34	Echo_pump_function	[%]
35	Echo_diastolic_dysfunction	
36	Echo_cava_stowed	
37	Echo_mitral_regurgitation	
38	Echo_mitral_stenosis	
39	Echo_aortic_regurgitation	
40	Echo_aortic_stenosis	
41	Ao_LA_ratio	
42	Septum hypertrophy	
43	Posterior wall hypertrophy	
44	Non concentric hypertrophy	
45	Ejection fraction teicholz	
46	Teicholz >/< 45%	
